# EV71 vaccines: a milestone in the history of global vaccine development

**DOI:** 10.1038/emi.2014.29

**Published:** 2014-04-16

**Authors:** Shan Lu

**Affiliations:** Department of Medicine, University of Massachusetts Medical School, Worcester, MA 01605, USA

**Three vaccines against enterovirus 71 (EV71) have completed phase 3 clinical trials with good safety and efficacy results. These vaccines may benefit children living in Asian countries who have been under the threat of severe hand, foot and mouth disease in recent years. These vaccines may also represent the start of a new trend for more vaccines against emerging infections in developing countries. A global system that includes contributions from vaccine producers in developing countries is needed to ensure the safety, quality and access of these new vaccines.**

From May 2013 to February 2014, three vaccine producers from China published phase 3 clinical trial results demonstrating the efficacy of three inactivated vaccines against human enterovirus 71 (EV71).^1,2,3^ Overall, the safety profiles of these vaccines were good, and extremely high levels of protection were elicited (Table 1).

EV71 and coxsackievirus 16 (CA16) are two leading causes of hand, foot and mouth disease (HFMD), which is a common childhood exanthema. Both viruses are members of the human enterovirus genus of the *Picornaviridae* family. While symptoms in most cases of HFMD are mild, HFMD cases caused by EV71 carry higher risk of severe complications, including neurologic disorders and even death.

EV71 was first discovered in North America close to one half century ago and has since been found in many parts of the world. Only after the late 1990s did EV71 become a major emerging infectious disease in Asia. Large EV71 epidemics with significant numbers of fatalities have been reported in Malaysia, Taiwan, Mainland China, and other Southeast and East Asian countries. As indicated in an attempt to alert other parts of the world, my review published in 2010 suggested that EV71 is an emerging infectious disease vaccine target.^4^ However, few could have expected such rapid development of not just one but three vaccines in a short time frame.

Not all three phase 3 trials measured the control of severe HFMD-associated diseases given the low incidence of these complications; an effective vaccine to control EV71 infection should in theory also reduce the number of severe cases. Because EV71 is the primary pathogen known to cause severe cases of HFMD, there is no reason (at least for now) to expect any negative impact if there is a potential shift of instances of HFMD being caused by other enteroviruses due to the control of EV71.

As my colleagues and I questioned 4 years ago, it is not clear whether any of the major global vaccine companies will be interested in producing EV71 vaccines given that emerging EV71 infections are found primarily in regions outside the developed world and given that there are issues associated with the financing, intellectual property, regulatory review, clinical trials and market distribution of such vaccines. Some have hoped that major global vaccine companies would take the lead and collaborate with local vaccine manufacturers to develop such vaccines. However, in light of the issues listed above and speculation as to whether EV71 infections will continue or if they will occur only as random outbreaks, these major vaccine players did not establish EV71 vaccine programs.

The timing is right for China-based vaccine producers. Approximately one decade ago, there was a non-official “decentralization” in China's vaccine industry that resulted in the establishment of multiple private vaccine companies. This created direct competition against the traditional state-owned Biological Products Institutes, which also went through major changes to survive in the marketplace. Challenged by a number of major infectious diseases in the last decade, including severe acute respiratory syndrome, avian influenza and pandemic H1N1 influenza, both private and public vaccine companies in China have grown significantly by investing heavily in scale, technology, and matching processes to develop and test candidate vaccines in human studies. Three EV71 vaccines as reported were manufactured by either private or public vaccine producers.

Several lessons can be drawn from the rapid development of EV71 vaccines. First, funding from the central Chinese government played a key role in major infectious disease research and development programs in the last decade. Multiple funding mechanisms exist to support different phases of vaccine product development. Second, a traditional technology, the inactivated vaccine, was used to develop these EV71 vaccines. Chinese vaccine producers have relied on this approach to develop candidate vaccines against multiple emerging infections, such as avian and pandemic influenza viruses. Given the success of inactivated vaccines against poliovirus, which is another member of the *Picornaviridae* family, it is not totally surprising that an inactivated vaccine can work against EV71. Many unknown questions associated with a new vaccine technology can be avoided by using a well-established vaccine platform. Third, there was a close collaboration among vaccine producers, groups from key local Centers for Disease Control and Prevention (which run clinical trials), and the National Institutes of Food and Drug Control, as evidenced by co-authorship among personnel from these groups on the published reports of the phase 3 results. There will no doubt be questions on how to set rules to avoid conflicts of interest, but this unusual system contributed to the rapid development of a product with significant national interest.

The reports of three EV71 vaccines completing phase 3 trials represent a milestone in the history of the global vaccine field. Only a few first-in-man vaccines have been completely developed outside Western countries. In recent years, China has licensed the first ever hepatitis E virus (HEV) vaccine.^5^ The phase 3 HEV vaccine study was even larger than the EV71 vaccine clinical trials, with over 112 000 volunteers in the trial. Such a scale of operation will become increasingly necessary due to the nature of emerging infections but will also prevent even the most profitable vaccine companies in the world from taking these projects. Recently, several major global pharmaceutical companies have either scaled down their vaccine divisions or are in the process of divesting the vaccine business. One of the key challenges that they could not address was how to develop vaccines against biodefense or emerging infectious disease targets that may not be applicable to an entire nation or are only required in certain regions of the world.

It would be naïve to assert that vaccine developers from developing countries will dominate the world vaccine market, and it is unclear whether Chinese vaccine producers can sustain a massive scale of new vaccine development. While EV71 vaccines are successful, based on published reports, more questions remain. For instance, should such vaccines be added to China's national vaccination schedule, and should vaccines against other leading viruses for HFMD, such as CA16, be included as part of the formulation? Will Chinese vaccine producers be interested and able to provide EV71 vaccines to other countries in Asia to further control regional HFMD epidemics? Furthermore, will these vaccines generate cross-protection against the EV71 virus of other genotypes, as reported with another candidate EV71 vaccine under development?^6^ Additionally, information on EV71 vaccine-induced immunity remains limited. Samples collected during phase 3 efficacy trials should be analyzed for immune response types and immune correlates of protection. Answers to such questions are important to further ensure the wide and safe use of EV71 vaccines both inside and outside China.

The successful production of EV71 vaccines, in addition to the previous HEV vaccine, may mark a turning point for future global vaccine development. Chinese vaccine producers should be proud of their contribution to the world. However, they need to move up the technology ladder to develop products beyond well-established vaccine approaches. Enhanced pre- and post-marketing safety standards and monitoring systems, as well as tailored public education, should be developed, especially in light of recent public concerns of the safety of licensed vaccine products that have long been available. The global vaccine community, including major vaccine companies, should embrace this shift by seeking new opportunities to work with vaccine producers from developing countries. It is likely that most future vaccine targets will not be as easy as EV71. Technology, global collaboration, and public support will drive the future of vaccine development.

## Figures and Tables

**Table 1:** Summary of key features of three EV71 vaccines^1,2,3^

Vaccine producer	Beijing Vigoo Biological	Sinovac Biotech	Institute of Medical Biology, CAMS^a^
Total trial population	10245	10007	12000
Age (month)	6–35	6–35	6–71
Dosing	320 U	400 U	100 U
Immunization schedule (day)	0, 28	0, 28	0, 28
Adjuvant	Alum 0.18 mg	Alum - b	Alum 0.5 mg
Viral strain	FY7VP5/AH/CHN/2008 (genotype C4)	EV71 strain H07 (genotype C4)	An EV71 strain of genotype C4 isolated from Fuyan, China
Vaccine efficacy (against EV71-associated HFMD)	90.0%	94.8%	97.3%–97.4%

a Chinese Academy of Medical Sciences, CAMS.

b Alum dosing information is not included in Reference 2.
